# Minimum capital requirement and portfolio allocation for non-life insurance: a semiparametric model with Conditional Value-at-Risk (CVaR) constraint

**DOI:** 10.1007/s10287-023-00439-1

**Published:** 2023-03-03

**Authors:** Alessandro Staino, Emilio Russo, Massimo Costabile, Arturo Leccadito

**Affiliations:** 1 Department of Economics, Statistics and Finance, University of Calabria, Ponte Bucci cubo 1C, 87036 Rende (CS), Italy; 2 LFIN/LIDAM, UCLouvain, Voie du Roman Pays 34, 1348 Louvain la neuve, Belgium

**Keywords:** Non-life insurance, Capital requirement, Conditional Value-at-Risk, Convex optimization

## Abstract

We present an optimization problem to determine the minimum capital requirement for a non-life insurance company. The optimization problem imposes a non-positive Conditional Value-at-Risk (CVaR) of the insurer’s net loss and a portfolio performance constraint. When expressing the optimization problem in a semiparametric form, we demonstrate its convexity for any integrable random variable representing the insurer’s liability. Furthermore, we prove that the function defining the CVaR constraint in the semiparametric formulation is continuously differentiable when the insurer’s liability has a continuous distribution. We use the Kelley-Cheney-Goldstein algorithm to solve the optimization problem in the semiparametric form and show its convergence. An empirical analysis is carried out by assuming three different liability distributions: a lognormal distribution, a gamma distribution, and a mixture of Erlang distributions with a common scale parameter. The numerical experiments show that the choice of the liability distribution plays a crucial role since marked differences emerge when comparing the mixture distribution with the other two distributions. In particular, the mixture distribution describes better the right tail of the empirical distribution of liabilities with respect to the other two distributions and implies higher capital requirements and different assets in the optimal portfolios.

## Introduction

Establishing an initial capital that insurance companies must hold to protect themselves from unexpected events over a fixed period is a primary goal of insurance regulation. The computation of this initial capital relies on a risk measure. For instance, the Solvency II directive (see Directive 2009/138/EC), which regulates the solvency capital requirement of the insurance companies in the European Union, adopts the Value-at-Risk (VaR) as a risk measure, even if, as evidenced by Artzner et al. (1999), it is not a coherent risk measure because it does not fulfil the sub-additivity property. Unlike VaR, the Conditional Value-at-Risk (CVaR), or Expected Shortfall, adopted by the Swiss Solvency Test (see Federal Office of Private Insurance (FOPI) 2004), represents a coherent risk measure and, as showed by Artzner (1999) and Acerbi and Tasche (2002), it is a more reliable risk measure than the VaR for evaluating the risk of financial positions or computing capital requirements. Supporting this evidence, Dhaene et al. (2006) provided a comprehensive analysis of the theoretical properties of well-known risk measures that can be applied in the context of solvency capital requirements. They also gave special attention to the class of distortion risk measures that includes the CVaR as a particular case, and they investigated the relationship between these risk measures and theories of choice under risk.

When connecting minimum capital requirements to risk measures, the standard approach consists in investing the minimum capital in a single security, which is often a risk-free asset. However, Balbás (2008) showed the non-optimality of such a strategy in many important cases, and Farkas et al. (2015) laid out new results for risk measures when investing in multiple eligible assets. The assumption of investing the minimum capital in a portfolio of assets implies the definitions of optimization problems that employ both the initial capital and portfolio weights as decision variables. In this sense, Mankai and Bruneau (2012) proposed an optimization problem with a CVaR constraint that maximizes the expected return on risk-adjusted capital and uses both the initial capital and portfolio weights as decision variables. Asimit et al. (2015) developed an optimization problem that minimizes the initial capital and optimally allocate it among the available financial assets under a ruin probability constraint. In this research line, Asanga et al. (2014) defined three optimization problems representing a dynamic improvement of the approach developed in Asimit et al. (2015). The first problem uses a ruin probability constraint as in Asimit et al. (2015), the second problem a CVaR constraint, and the third problem an expected policyholder deficit constraint. All the three problems include a portfolio performance constraint defined as a lower bound on the expected return on capital (ROC). Kaucic and Daris (2015) proposed an alternative approach to deal with multi-objective portfolio optimization problems with chance constraints and applied this optimization framework to an EU-based non-life insurance company that tries to minimize the risk of the discrepancy between assets and liabilities. The authors adopt shareholders’ capital and investment weights as decision variables.

In the present paper, we focus on the optimization problem with the CVaR constraint of Asanga et al. (2014). The standard approach for solving the problem relies on a Monte Carlo approximation that leads to a linear programming (LP) formulation (see Rockafellar and Uryasev 2000, 2002; Krokhmal et al. 2002), whose solution becomes computationally burdensome when the number of simulations is large. To tackle this issue, Asanga et al. (2014) proposed a semiparametric formulation showing its convexity when the insurer’s liability is lognormally distributed. Deepening this evidence, we find that the CVaR optimization problem in the semiparametric form is convex for any integrable liability distribution. Furthermore, when the insurer’s liability has a continuous distribution, the function defining the CVaR constraint is continuously differentiable allowing us to obtain its gradient. To solve the considered problem, we use the Kelley-Cheney-Goldstein (KCG) algorithm and show its convergence to an optimal solution.

We apply the CVaR optimization problem assuming three different liability distributions: a lognormal distribution, a gamma distribution, and a mixture of Erlang distributions with a common scale parameter. The lognormal and gamma distributions are unimodal distributions commonly used to describe insurance data. However, insurance data often present a multimodal shape and fatter tails than lognormal or gamma distributions. Hence, mixtures of Erlang distributions with a common scale parameter are more suitable for capturing such features (see, e.g., Lee and Lin 2010; Willmot and Lin 2011; Lee and Lin 2012; Verbelen et al. 2015). This class of distributions is dense in the space of positive continuous distributions (see Tijms 1994) and hence allows us to approximate any positive continuous distribution. Moreover, we can easily compute risk measures, such as VaR and CVaR, and aggregate risks because mixtures of Erlang distributions with a common scale parameter are closed under convolution and compounding (see Lee and Lin 2010, and the references therein for further properties of this class of distributions).

To conduct the numerical experiments, we use the insurer’s liability data set *danishuni* from the R package *CASdatasets* of Dutang and Charpentier (2020) comprising 2167 fire losses from January 1980 to December 1990. We adjust this data set using the U.S. inflation to reflect losses from January 2010 to December 2020. Regarding the assets, we use daily log-returns of the S&P 500 index and two exchange-traded funds that track the investment results of U.S. treasury and corporate bond indices. The numerical experiments show that the choice of the liability distribution plays a crucial role, and the most marked differences are between the mixture distribution and the other two distributions. Indeed, since the mixture distribution can describe the right tail of the empirical distribution of the insurer’s liability better than the other two distributions, it implies higher capital requirements. The differences among the three liability distributions depend on the available liability data set. If the empirical distributions are unimodal and with a not-too-fat right tail, the differences among the capital requirements computed with the three liability distributions are less evident. On the contrary, when the empirical distributions are multimodal, or their right tail is fat, mixtures of Erlang distributions with a common scale parameter are more suitable to the scope of capturing accurately the data distribution, and marked differences in the computed capital requirements arise in comparison to the other two distributions. This is a crucial aspect to investigate since, as evidenced by Lee and Lin (2010), actuaries often face heavy-tailed and/or irregular data.

The paper makes the following contributions. Firstly, we prove that the optimization problem in the semiparametric form is convex for any integrable insurer’s liability and that the function defining the CVaR constraint under the semiparametric formulation is continuously differentiable when the insurer’s liability has a continuous distribution. Secondly, we show how to implement the KCG algorithm to solve the optimization problem in the semiparametric form, and we prove its convergence to an optimal solution. Lastly, we are, to the best of our knowledge, the first to use the mixture of Erlang distributions to describe the insurer's liability in the context of capital requirement computation.

The rest of the article is organized as follows. In Sect. 2, after recalling the optimization problem with CVaR constraint and portfolio performance constraint of Asanga et al. (2014), we show how to obtain the semiparametric formulation. Furthermore, we derive some theoretical results regarding the optimization problem in the semiparametric form. We conclude Sect. 2 by reporting the KCG algorithm for solving the optimization problem in the semiparametric form and giving a proposition that states the algorithm convergence. In Sect. 3, we explain the method for generating asset log-return scenarios that we use in the semiparametric formulation of the optimization problem and show the form of the CVaR constraint in relation to the three distributions used to model the insurer’s liability. The empirical results with an in-sample and out-of-sample analysis are shown in Sect. 4. Finally, in Sect. 5, we conclude the article.

## Optimization with CVaR constraint

Given a probability space $\left( \Omega ,\mathcal {F},\mathbb {P}\right)$ and a set $\mathcal {T}=\{0,1,\ldots ,T\}$ of trading dates, we consider a financial market made up of *n* assets with gross returns over the period $[t,t+1]$ being the components of the random vector ${\textbf {R}}_{t+1}=(R_{1,t+1},\ldots ,R_{n,t+1})'$. We denote by $\mathcal {F}_t$ the historical information on the asset gross return evolution up to time *t*, that is, $\mathcal {F}_t=\sigma ({\textbf {R}}_{1},\ldots ,{\textbf {R}}_{t})$, and set $\mathbb {P}_t(\cdot )=\mathbb {P}(\cdot \vert \mathcal {F}_t)$ and $E_t[\cdot ]=E[\cdot \vert \mathcal {F}_t]$.

The optimization problem at a generic time $t\in \mathcal {T}$ involves a non-life insurance company with a one-period setting $[t,t+\tau ]$, where $\tau$ is an integer representing the solvency horizon, $\tau \le T-t$. We denote by $p_t$ the premium collected from policyholders and available for investment at time *t* and suppose that the insurer provides a regulatory initial capital of size $c_t$, i.e., $p_t+c_t$ is the total amount that the insurer invests in *t*. The vector ${\textbf {x}}_t=(x_{1,t},\ldots ,x_{n,t})'$ contains the portfolio weights at time *t*, and it satisfies the budget constraint $\sum _{i=1}^nx_{i,t}=1$ with no short sales allowed, i.e., $x_{i,t}\ge 0$, for $i=1,\ldots ,n$.

The univariate random variable (r.v.) $Y_{t,t+\tau }$ represents the insurer’s liability over the period $[t,t+\tau ]$, and we assume that the payment of $Y_{t,t+\tau }$ occurs in $t+\tau$. Then, the insurer’s net loss over the solvency horizon is

$$\begin{aligned} L_{t,t+\tau }=Y_{t,t+\tau }-(p_t+c_t){\textbf {R}}_{t,t+\tau }'{} {\textbf {x}}_t, \end{aligned}$$

where ${\textbf {R}}_{t,t+\tau }$ denotes the gross return vector over $[t,t+\tau ]$ with the *i*th component equal to $\prod _{\iota =1}^\tau R_{i,t+\iota }$.

The optimization problem has the portfolio weights ${\textbf {x}}_t$ and the capital requirement $c_t$ as decision variables and minimizes $c_t$ subject to two key constraints. One constraint is the solvency constraint that imposes a non-positive CVaR for $L_{t,t+\tau }$. Given the confidence level $\alpha \in (0,1)$, the CVaR of $L_{t,t+\tau }$ in *t* is defined as

$$\begin{aligned} \text {CVaR}_t^\alpha (L_{t,t+\tau })=\frac{1}{1-\alpha }\int _\alpha ^1\text {VaR}_t^\beta (L_{t,t+\tau })\,d\beta , \end{aligned}$$

where

$$\begin{aligned} \text {VaR}_t^\beta (L_{t,t+\tau })=\inf \left\{ l\in \mathbb {R}:\mathbb {P}_t(L_{t,t+\tau }\le l)\ge \beta \right\} \end{aligned}$$

is the VaR of $L_{t,t+\tau }$ in *t* at level $\beta$. This definition of CVaR corresponds to that given in Kaas et al. (2008), where the name Tail-Value-at-Risk is used instead of CVaR. The CVaR of $L_{t,t+\tau }$ in *t* can also be expressed as

$$\begin{aligned} \text {CVaR}_t^\alpha (L_{t,t+\tau })=\text {VaR}_t^\alpha (L_{t,t+\tau })+\frac{1}{1-\alpha }E_t\left[ \left( L_{t,t+\tau }-\text {VaR}_t^\alpha (L_{t,t+\tau })\right) _{+}\right] , \end{aligned}$$

where the positive part is defined as $(x)_+=\max (x,0)$, and it holds the characterization

1 $$\begin{aligned} \text {CVaR}_t^\alpha (L_{t,t+\tau })=\inf _{s\in \mathbb {R}}\left\{ s+\frac{1}{1-\alpha } E_t\left[ \left( L_{t,t+\tau }-s\right) _{+}\right] \right\} . \end{aligned}$$

The second constraint is a portfolio performance constraint defined as

$$\begin{aligned} E_t[\text {ROC}_{t,t+\tau }]\ge \gamma , \end{aligned}$$

where

$$\begin{aligned} \text {ROC}_{t,t+\tau }=-\frac{L_{t,t+\tau }}{c_t} \end{aligned}$$

is the return on capital over the period $[t,t+\tau ]$ and $\gamma$ is the associated lower bound. Hence, the optimization problem with CVaR solvency constraint is given by

2 $$\begin{aligned} \min _{c_t,{\textbf {x}}_t}&\quad c_t \end{aligned}$$

3 $$\begin{aligned} \quad \text {s.t.}&\quad \text {CVaR}_t^\alpha (L_{t,t+\tau })\le 0, \end{aligned}$$

4 $$\begin{aligned}&\quad E_t[\text {ROC}_{t,t+\tau }]\ge \gamma , \end{aligned}$$

5 $$\begin{aligned}&\quad {\textbf {1}}'{} {\textbf {x}}_t=1,\;{\textbf {x}}_t\ge {\textbf {0}},\;c_t\ge 0, \end{aligned}$$

where ${\textbf {0}}$ and ${\textbf {1}}$ are vectors of size *n* with all the elements equal to 0 and 1, respectively, and ${\textbf {x}}_t\ge {\textbf {0}}$ means that the inequality must hold element-by-element. By equation (1), Problem (2)−(5) has the equivalent formulation

6 $$\begin{aligned} \min _{s,c_t,{\textbf {x}}_t}&\quad c_t \end{aligned}$$

7 $$\begin{aligned} \quad \text {s.t.}&\quad s+\frac{1}{1-\alpha }E_t\left[ \left( L_{t,t+\tau }-s\right) _{+}\right] \le 0, \end{aligned}$$

8 $$\begin{aligned}&\quad E_t[\text {ROC}_{t,t+\tau }]\ge \gamma , \end{aligned}$$

9 $$\begin{aligned}&\quad {\textbf {1}}'{} {\textbf {x}}_t=1,\;{\textbf {x}}_t\ge {\textbf {0}},\;c_t\ge 0, \end{aligned}$$

and the traditional solution method relies upon approximating the conditional expectation of the CVaR constraint with a Monte Carlo-type estimator that leads to an LP problem. Indeed, if we generate *m* scenarios for $Y_{t,t+\tau }$ and ${\textbf {R}}_{t,t+\tau }$, namely $Y_{t,t+\tau }(j)$ and ${\textbf {R}}_{t,t+\tau }(j)$ with $j=1,\ldots ,m$, conditional on $\mathcal {F}_t$ and set ${\textbf {z}}_t=(p_t+c_t){\textbf {x}}_t$, Problem (6)–(9) becomes

$$\begin{aligned} \min _{s,c_t,{\textbf {z}}_t}&\quad c_t \nonumber \\ \quad \text {s.t.}&\quad s+\frac{1}{m(1-\alpha )}\sum _{j=1}^{m}\left( Y_{t,t+\tau }(j)-{\textbf {R}}_{t,t+\tau }'(j){\textbf {z}}_t-s\right) _{+}\le 0, \nonumber \\&\quad \frac{1}{m}\sum _{j=1}^m\left( {\textbf {R}}_{t,t+\tau }'(j){\textbf {z}}_t-Y_{t,t+\tau }(j)\right) \ge \gamma c_t, \nonumber \\&\quad {\textbf {1}}'{} {\textbf {z}}_t=p_t+c_t,\;{\textbf {z}}_t\ge {\textbf {0}},\;c_t\ge 0, \nonumber \end{aligned}$$

and it can be linearised by introducing the non-negative variables ${\textbf {y}}=(y_1,\ldots ,y_m)'$ as

$$\begin{aligned} \min _{s,c_t,{\textbf {z}}_t}&\quad c_t \nonumber \\ \quad \text {s.t.}&\quad s+\frac{1}{m(1-\alpha )}\sum _{j=1}^{m}y_j\le 0, \nonumber \\&\quad y_j\ge Y_{t,t+\tau }(j)-{\textbf {R}}_{t,t+\tau }'(j){\textbf {z}}_t-s,\quad j=1,\ldots ,m, \nonumber \\&\quad \frac{1}{m}\sum _{j=1}^m\left( {\textbf {R}}_{t,t+\tau }'(j){\textbf {z}}_t-Y_{t,t+\tau }(j)\right) \ge \gamma c_t \nonumber \\&\quad {\textbf {y}}\ge {\textbf {0}},\;{\textbf {1}}'{} {\textbf {z}}_t=p_t+c_t,\;{\textbf {z}}_t\ge {\textbf {0}},\;c_t\ge 0. \nonumber \end{aligned}$$

When *m* is too large, solving this LP formulation becomes computationally burdensome, and alternative approaches are necessary. For instance, Asanga et al. (2014) proposed the following semiparametric reformulation

10 $$\begin{aligned} \min _{s,c_t,{\textbf {z}}_t}&\quad c_t \end{aligned}$$

11 $$\begin{aligned} \quad \text {s.t.}&\quad s+\frac{1}{m(1-\alpha )}\sum _{j=1}^{m}E_t^{(j)}\left[ \left( Y_{t,t+\tau }-{\textbf {R}}_{t,t+\tau }'(j){\textbf {z}}_t-s\right) _{+}\right] \le 0, \end{aligned}$$

12 $$\begin{aligned}&\quad \frac{1}{m}\sum _{j=1}^m{\textbf {R}}_{t,t+\tau }'(j){\textbf {z}}_t-\mu _{t,t+\tau }\ge \gamma c_t, \end{aligned}$$

13 $$\begin{aligned}&\quad {\textbf {1}}'{} {\textbf {z}}_t=p_t+c_t,\;{\textbf {z}}_t\ge {\textbf {0}},\;c_t\ge 0, \end{aligned}$$

where $E_t^{(j)}[\cdot ]=E_t[\cdot \vert \mathcal {F}_t\bigvee \sigma \left( \{{\textbf {R}}_{t,t+\tau }={\textbf {R}}_{t,t+\tau }(j)\}\right) ]$ and $\mu _{t,t+\tau }=E_t[Y_{t,t+\tau }]$. The symbol $\bigvee$ denotes the smallest $\sigma$-algebra containing the two $\sigma$-algebras $\mathcal {F}_t$ and $\sigma \left( \{{\textbf {R}}_{t,t+\tau }={\textbf {R}}_{t,t+\tau }(j)\}\right)$. By defining the function

14 $$\begin{aligned} g(s,{\textbf {z}}_t)=s+\frac{1}{m(1-\alpha )}\sum _{j=1}^{m}E_t^{(j)}\left[ \left( Y_{t,t+\tau }-{\textbf {R}}_{t,t+\tau }'(j){\textbf {z}}_t-s\right) _{+}\right] , \end{aligned}$$

the CVaR constraint (11) can be equivalently written as $g(s,{\textbf {z}}_t)\le 0$. Problem (10)−(13) is convex because $g(s,{\textbf {z}}_t)$ is a convex function. Indeed, for the convexity of $g(s,{\textbf {z}}_t)$, it is sufficient to prove that the function

15 $$\begin{aligned} E_t^{(j)}\left[ \left( Y_{t,t+\tau }-{\textbf {R}}_{t,t+\tau }'(j){\textbf {z}}_t-s\right) _{+}\right] \end{aligned}$$

is convex in the variables *s* and ${\textbf {z}}_t$. Since the positive part is a convex function, we can easily verify that the function

$$\begin{aligned} h^{(j)}(l)=E_t^{(j)}\left[ \left( Y_{t,t+\tau }-l\right) _{+}\right] , \end{aligned}$$

is convex too. Then, the convexity of (15) follows because we have a convex function applied to a linear transformation (see, e.g., Theorem 5.7 in Rockafellar 1970).

To solve Problem (10)–(13), we use an iterative algorithm that, at each iteration, requires the gradient, or a subgradient, of the current solution for the CVaR constraint. Proposition 1 states that the CVaR constraint is continuously differentiable and provides the gradient of $g(s,{\textbf {z}}_t)$ if $Y_{t,t+\tau }$ has a continuous distribution.^1^

### Proposition 1

When the r.v. $Y_{t,t+\tau }$ is continuous with cumulative distribution function $F_{Y_{t,t+\tau }}$, the function $g(s,{\textbf {z}}_t)$ is continuously differentiable with gradient

$$\begin{aligned} \nabla g(s,{\textbf {z}}_t) =\begin{bmatrix} 1+\frac{1}{m(1-\alpha )}\sum _{j=1}^mw(j)\\ \frac{1}{m(1-\alpha )}\sum _{j=1}^m\left[ w(j)\prod _{k=1}^\tau R_{1,t+k}(j)\right] \\ \vdots \\ \frac{1}{m(1-\alpha )}\sum _{j=1}^m\left[ w(j)\prod _{k=1}^\tau R_{n,t+k}(j)\right] \end{bmatrix}, \end{aligned}$$

where

$$\begin{aligned} w(j)=-1+F_{Y_{t,t+\tau }}({\textbf {R}}_{t,t+\tau }'(j){\textbf {z}}_t+s), \end{aligned}$$

for $$j=1,\ldots ,m$$.

### Proof

Since $Y_{t,t+\tau }$ is a continuous r.v., Lemma 1 of Rockafellar and Uryasev (2000) ensures the differentiability of $h^{(j)}(l)=E_t^{(j)}\left[ \left( Y_{t,t+\tau }-l\right) _{+}\right]$ with derivative

$$\begin{aligned} \frac{d}{dl}h^{(j)}(l)=-1+F_{Y_{t,t+\tau }}(l). \end{aligned}$$

Then, the statement of the proposition about the gradient of the function $g(s,{\textbf {z}}_t)$ is just a consequence of the classical chain rule of multivariate calculus. The components of $\nabla g(s,{\textbf {z}}_t)$ are all continuous functions, hence the function $g(s,{\textbf {z}}_t)$ is continuously differentiable.

In general, for any integrable r.v. $Y_{t,t+\tau }$, the function $g(s,{\textbf {z}}_t)$ admits the subdifferential

$$\begin{aligned} \partial g(s,{\textbf {z}}_t) =\begin{bmatrix} 1+\frac{1}{m(1-\alpha )}\sum _{j=1}^mw(j)\\ \frac{1}{m(1-\alpha )}\sum _{j=1}^m\left[ w(j)\prod _{k=1}^\tau R_{1,t+k}(j)\right] \\ \vdots \\ \frac{1}{m(1-\alpha )}\sum _{j=1}^m\left[ w(j)\prod _{k=1}^\tau R_{n,t+k}(j)\right] \end{bmatrix}, \end{aligned}$$

where *w*(*j*) now represents the subdifferential of the function $h^{(j)}(l)=E_t^{(j)}\left[ \left( Y_{t,t+\tau }-l\right) _{+}\right]$ evaluated at point ${\textbf {R}}_{t,t+\tau }'(j){\textbf {z}}_t+s$. Specifically, *w*(*j*) is the interval [*a*, *b*] where

$$\begin{aligned} a=-1+\mathbb {P}(Y_{t,t+\tau }<{\textbf {R}}_{t,t+\tau }'(j){\textbf {z}}_t+s)\quad \text {and}\quad b=-1+\mathbb {P}(Y_{t,t+\tau }\le {\textbf {R}}_{t,t+\tau }'(j){\textbf {z}}_t+s). \end{aligned}$$

When the distribution of $Y_{t,t+\tau }$ does not have a probability mass at point ${\textbf {R}}_{t,t+\tau }'(j){\textbf {z}}_t+s$, the subdifferential $\partial g(s,{\textbf {z}}_t)$ is only composed of the gradient $\nabla g(s,{\textbf {z}}_t)$ with the expression specified in Proposition 2.

To solve Problem (10)−(13), we propose applying the Kelley-Cheney-Goldstein (KCG) algorithm (see Kelley 1960). We assume that it holds $(s,{\textbf {z}}_t)\in R=\{(s',{\textbf {z}}'):|s'|\le \lambda ,0\le z'_{1}\le \lambda ,\ldots ,0\le z'_{n}\le \lambda \}$, with $\lambda >0$ large enough, for each feasible point $(s,c_t,{\textbf {z}}_t)$. Then, the KCG algorithm for Problem (10)–(13) is Algorithm 1.
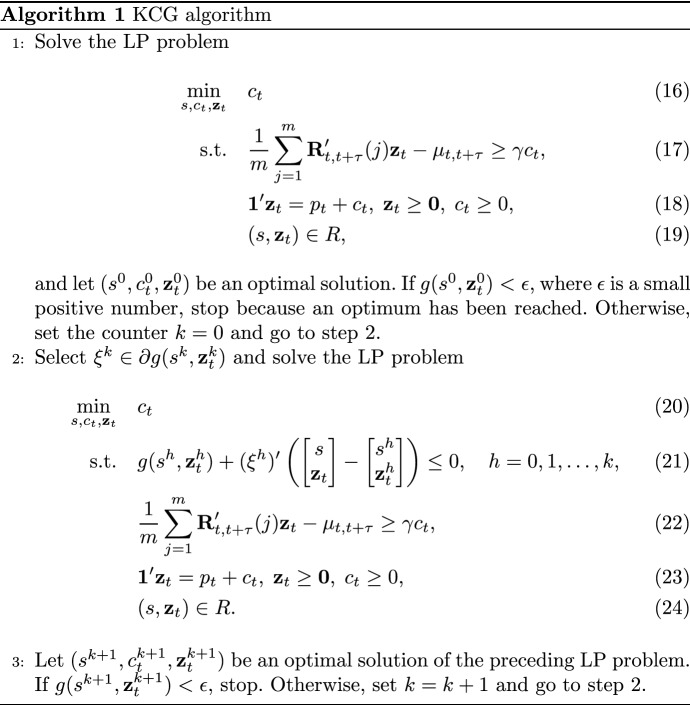


Proposition 2 states the convergence of the KCG algorithm for Problem (10)–(13).

### Proposition 2

Suppose that the feasible set $\mathcal {G}$ of Problem (10)–(13) is nonempty and that there exists a positive number $\lambda$ such that $(s,{\textbf {z}}_t)\in R=\{(s',{\textbf {z}}'):|s'|\le \lambda ,0\le z'_{1}\le \lambda ,\ldots ,0\le z'_{n}\le \lambda \}$ for each $(s,c_t,{\textbf {z}}_t)\in \mathcal {G}$. Furthermore, suppose that there exists a positive number *K* such that

$$\begin{aligned} \sup \{||\xi ||:\xi \in \partial g(s,{\textbf {z}}_t),\;(s,{\textbf {z}}_t)\in R\}\le K. \end{aligned}$$

Let $(s^0,c_t^0,{\textbf {z}}_t^0)$ be an optimal solution to Problem (16)–(19) and $(s^{k+1},c_t^{k+1},{\textbf {z}}_t^{k+1})$ an optimal solution to Problem (20)–(24), then the sequence $\{(s^k,c_t^k,{\textbf {z}}_t^k)\}$ contains a subsequence converging to an optimal solution for Problem (10)–(13).

### Proof

Let $S_0$ denote the feasible set of Problem (16)–(19) and $S_{k+1}$ the feasible set of Problem (20)–(24), then

$$\begin{aligned} \mathcal {G}=S_0\cap G, \end{aligned}$$

where $$G=\{(s,c_t,{\textbf {z}}_t):g(s,{\textbf {z}}_t)\le 0\}$$, and

16 $$\begin{aligned} \mathcal {G}\subset S_{k+1},\quad \forall k\ge 0, \end{aligned}$$

because, by definition of subgradient (see, e.g., Section 23 in Rockafellar 1970),

$$\begin{aligned} g(s,{\textbf {z}}_t)\ge g(s^k,{\textbf {z}}_t^k)+(\xi ^k)'\left( \begin{bmatrix} s\\ {\textbf {z}}_t \end{bmatrix}-\begin{bmatrix} s^k\\ {\textbf {z}}_t^k \end{bmatrix}\right) ,\quad \forall (s,{\textbf {z}}_t)\in R, \end{aligned}$$

where $$(s^k,{\textbf {z}}_t^k)\in R$$ and $$\xi ^k\in \partial g(s^k,{\textbf {z}}_t^k)$$.

We split the sequence $\{(s^k,c_t^k,{\textbf {z}}_t^k)\}$ into the two sequences $\{c_t^k\}$ and $\{(s^k,{\textbf {z}}_t^k)\}$. Since $S_{k-1}\subset S_{k-2}\subset \cdots \subset S_0$, and the decision variable $c_t$ corresponds to the objective function in Problem (10)–(13), the sequence $\{c^k_t\}$ is monotone increasing. Moreover, the sequence $\{c_t^k\}$ is bounded since

$$\begin{aligned} 0\le c_t^k={\textbf {1}}'{} {\textbf {z}}_t^k-p_t\le n\lambda -p_t,\;\forall k, \end{aligned}$$

and we conclude that $\{c^k_t\}$ converges to a point $c^*$. Hence, if $\{(s^k,{\textbf {z}}^k_t)\}$ contains a subsequence $\{(s^{k_j},{\textbf {z}}^{k_j}_t)\}$ converging to a point $(s^*,{\textbf {z}}^*)\in G'=\{(s,{\textbf {z}}_t):g(s,{\textbf {z}}_t)\le 0\}$, then $\{(s^{k_j},c^{k_j}_t,{\textbf {z}}^{k_j}_t)\}$ converges to $(s^*,c^*,{\textbf {z}}^*)$ that solves Problem (10)–(13). Indeed, the point $(s^*,c^*,{\textbf {z}}^*)$ belongs to $\mathcal {G}$ because $(s^*,c^*,{\textbf {z}}^*)\in G$ and it is an accumulation point for the compact set $S_0$, so that $(s^*,c^*,{\textbf {z}}^*)\in S_0$. Moreover, there could not exist another point $(s',c',{\textbf {z}}')\in \mathcal {G}$ with $c'<c^*$ because, otherwise, by the convergence of the monotone increasing sequence $\{c_t^{k_j}\}$ to $c^*$, there would exist an index *i* such that $c_t^{k_j}>c'$ for each $j>i$, thus contradicting the relation in (16).

Suppose now that $\{(s^k,{\textbf {z}}^k_t)\}$ does not have a subsequence converging to a point in $G'$. Then, there exists an $\alpha >0$, independent of *k*, such that

$$\begin{aligned} g(s^h,{\textbf {z}}_t^h)\ge \alpha , \end{aligned}$$

for $h=0,1,\ldots ,k$. If $(s^{k+1},c_t^{k+1},{\textbf {z}}_t^{k+1})$ solves Problem (20)–(24), then

$$\begin{aligned} g(s^h,{\textbf {z}}_t^h)+(\xi ^h)'\left( \begin{bmatrix} s^{k+1}\\ {\textbf {z}}_t^{k+1} \end{bmatrix}-\begin{bmatrix} s^h\\ {\textbf {z}}_t^h \end{bmatrix}\right) \le 0, \end{aligned}$$

for $h=0,1,\ldots ,k$. From the last two relations and the Cauchy-Schwarz inequality, it follows that

$$\begin{aligned} \alpha \le g(s^h,{\textbf {z}}_t^h)\le (\xi ^h)'\left( \begin{bmatrix} s^{h}\\ {\textbf {z}}_t^{h} \end{bmatrix}-\begin{bmatrix} s^{k+1}\\ {\textbf {z}}_t^{k+1} \end{bmatrix}\right) \le K\left| \left| (s^{h},{\textbf {z}}_t^{h})-(s^{k+1},{\textbf {z}}_t^{k+1})\right| \right| , \end{aligned}$$

for $h=0,1,\ldots ,k$. Hence, for every subsequence $\{k_j\}$ of indices, we have

$$\begin{aligned} \left| \left| (s^{k_j},{\textbf {z}}_t^{k_j})-(s^{k_i},{\textbf {z}}_t^{k_i})\right| \right| \ge \frac{\alpha }{K},\quad i<j, \end{aligned}$$

that is, $\{(s^k,{\textbf {z}}^k_t)\}$ does not have any Cauchy subsequence and this aspect contradicts that $\{(s^k,{\textbf {z}}^k_t)\}\subset R$ is bounded.

## Generation of asset log-return scenarios and liability modelling

To generate asset log-return scenarios, we use a moment-matching method. Given a sample of daily log-returns, we compute sample means, standard deviations, skewness, kurtosis, and correlations denoted by $\mu$, $\sigma$, $\delta$, $\kappa$, and $\rho$, respectively. Assuming independent and stationary increments, we use the following formulae for sample statistics referring to a different time scale:

26 $$\begin{aligned} \mu _\tau&=\tau \,\mu , \end{aligned}$$

27 $$\begin{aligned} \sigma _\tau&=\sqrt{\tau }\,\sigma , \end{aligned}$$

28 $$\begin{aligned} \delta _\tau&=\frac{\delta }{\sqrt{\tau }}, \end{aligned}$$

29 $$\begin{aligned} \kappa _\tau&=3\frac{\tau -1}{\tau }+\frac{\kappa }{\tau }, \end{aligned}$$

30 $$\begin{aligned} \rho _\tau&=\rho , \end{aligned}$$

where $\tau$ is the number of days in the new time scale. Given these target moments and correlations, to generate scenarios of log-returns over the period $[t,t+\tau ]$, we use the moment-matching method of Høyland et al. (2003) that ensures the matching of the first four moments for the marginal distributions and the matching of all the correlations. If ${\textbf {V}}_{t,t+\tau }(j)$ denotes the *j*th log-return scenario, then ${\textbf {R}}_{t,t+\tau }(j)=\exp ({\textbf {V}}_{t,t+\tau }(j))$ is the *j*th gross return scenario, where the exponential function is applied element-by-element. To evidence the differences with respect to the Asanga et al. (2014) approach, in Appendix B, we present an analysis where the generation of monthly log-returns is carried out with a multivariate generalized autoregressive conditional heteroskedastic (MV-GARCH) model, in particular, with the dynamic conditional correlation (DCC) model introduced by Engle (2002).

We model historical insurance data through three different continuous distributions: the lognormal distribution, the gamma distribution, and a mixture of Erlang distributions with a common scale parameter. The first two probability distributions are usually employed to fit historical data but they are unimodal, while empirical distributions show multimodal behaviour and fatter tails. In this sense, mixtures of Erlang distributions with a common scale parameter are more appropriate to capture the shape of empirical distributions. Moreover, Tijms (1994) proved that this class of distributions is dense in the space of positive continuous distributions and can be used to approximate any positive continuous distribution.

We report in Appendix A the definitions of the three probability distributions applied to describe the insurer’s liability. Here, we express the function *g* of Eq. (14) as

$$\begin{aligned} g(s,{\textbf {z}}_t)=s+\frac{1}{m(1-\alpha )}\sum _{j=1}^mh\left( {\textbf {R}}_{t,t+\tau }'(j){\textbf {z}}_t+s\right) \end{aligned}$$

and give the expression of $h(\cdot )$ corresponding to each liability distribution:

- If $Y_{t,t+\tau }$ is lognormally distributed with parameters $\mu \in \mathbb {R}$ and $\sigma >0$, then
  $$\begin{aligned} \begin{aligned} h(l)=&\left\{ \begin{aligned}&\exp \left( \mu +\frac{\sigma ^2}{2}\right) -l,&\text {if }l\le 0,\\&\exp \left( \mu +\frac{\sigma ^2}{2}\right) \Phi \left( \frac{\mu -\ln (l)+\sigma ^2}{\sigma }\right) -l\Phi \left( \frac{\mu -\ln (l)}{\sigma }\right) ,&\text {if }l>0, \end{aligned}\right. \end{aligned} \end{aligned}$$
  where $\Phi (\cdot )$ denotes the cumulative distribution function of a standard normal distribution;
- If $Y_{t,t+\tau }$ is gamma distributed with shape parameter $\beta >0$ and scale parameter $\theta >0$, then
  $$\begin{aligned} \begin{aligned} h(l)=&\left\{ \begin{aligned}&\beta \theta -l,&\text {if }l\le 0,\\&\beta \theta \left[ 1-F_G(l;\beta +1,\theta )\right] -l\left[ 1-F_G(l;\beta ,\theta )\right] ,&\text {if }l>0, \end{aligned}\right. \end{aligned} \end{aligned}$$
  where $F_G(\cdot ;\beta ,\theta )$ denotes the cumulative distribution function of a gamma r.v. with shape parameter $\beta$ and scale parameter $\theta$;
- if $Y_{t,t+\tau }$ is a mixture of $\varpi$ Erlang distributions with shape parameters $\beta _1,\ldots ,\beta _{\varpi }$ and a common scale parameter $\theta >0$, then
  $$\begin{aligned} \begin{aligned} h(l)=&\left\{ \begin{aligned}&\theta \sum _{i=1}^\varpi \alpha _i \beta _i-l,&\text {if }l\le 0,\\&\sum _{i=1}^\varpi \alpha _i\left\{ \beta _i\theta \left[ 1-F_G(l;\beta _i+1,\theta )\right] -l\left[ 1-F_G(l;\beta _i,\theta )\right] \right\} ,&\text {if }l>0, \end{aligned}\right. \end{aligned} \end{aligned}$$
  where $\alpha _1,\ldots ,\alpha _{\varpi }$ are the mixture weights, and $F_G(\cdot ;\beta ,\theta )$ denotes, as before, the cumulative distribution function of a gamma distribution with shape parameter $\beta$ and scale parameter $\theta$.

## Empirical analysis

In this section, we present an empirical analysis to show the differences that emerge when applying the optimization problem with CVaR constraint of Sect. 2 under the three liability distributions considered in Sect. 3. To generate asset log-returns, as explained in Sect. 3, we use the moment-matching method of Høyland et al. (2003). Similarly to Asanga et al. (2014), we perform both an efficient frontier analysis and an out-of-sample analysis. The additional analysis provided in Appendix B is an out-of-sample analysis with a DCC-GARCH model that allows us to investigate if a model with time-varying conditional volatilities and correlations (like the one used in Asanga et al. 2014) entails significant differences in the capital requirements and portfolio allocations with respect to the moment-matching method of Høyland et al. (2003).

### Data description

We consider portfolios composed of the S&P 500 index, the iShares Barclays 1-3 Year Treasury Bond ETF (SHY), and the iShares iBoxx $ Investment Grade Corporate Bond ETF (LQD).

**Table 1:** Descriptive statistics about daily log-returns from January 2010 to December 2020 for the assets S&P 500, SHY, and LQD

Asset	Min.	Max.	Mean	S.D.	Skewness	Kurtosis
S&P 500	–0.12765	0.08968	0.00043	0.01106	–0.86342	19.33641
SHY	–0.00439	0.00544	0.00005	0.00059	0.53278	9.60533
LQD	–0.05132	0.07131	0.00024	0.00448	0.32077	58.12501

**Table 2:** Descriptive statistics about the data set *danishuni* of the R package *CASdatasets* of Dutang and Charpentier (2020) after some adjustments to have monthly losses in millions of U.S. dollars and concerning the period January 2010 - December 2020

Min.	Max.	Mean	S.D.	Skewness	Kurtosis
3.59614	69.15245	13.88274	9.52692	3.50261	19.23287

We download daily adjusted closing prices from January 2010 to December 2020, for a total of 2768 observations, and to this aim we use the function *getSymbols* of the R package *quantmod* of Ryan and Ulrich (2022). As the source database, we download the asset prices from the website *Yahoo! Finance*. Table 1 reports descriptive statistics about the daily log-returns. We observe that S&P 500 has negative skewness and that LQD has the largest kurtosis. We split the computed daily log-returns into two samples, Sample *A* and Sample *B*. Sample *A* contains the observations from January 2010 to December 2015, that is, over the first six years, and we use it for the efficient frontier analysis. Sample *B* contains the remaining observations over the last five years, from January 2016 to December 2020, and we use it for the out-of-sample analysis.

Regarding the insurer’s liability, we use the data set *danishuni* of the R package *CASdatasets* of Dutang and Charpentier (2020) that comprises 2167 fire losses in millions of Danish krone from January 1980 to December 1990 adjusted for inflation to reflect year-1985 values. We convert these values in millions of U.S. dollars according to the exchange rate registered on 31 December 1985, which is 0.11198, aggregate the losses every month, and apply the annual inflation index so that the first monthly loss reflects the January 2010 value, the second monthly loss the February 2010 value, and so on, up to the last monthly loss that reflects the December 2020 value. Like the asset log-returns, we split these monthly losses into two samples. Sample $A'$ consists of monthly losses from January 2010 to December 2015, whereas Sample $B'$ consists of monthly losses from January 2016 to December 2020. Table 2 reports descriptive statistics for the data set *danishuni* after the adjustments detailed above.

**Table 3:** Parameters estimates of the three distributions taken into account for Sample $A'$. The losses in Sample $A'$ are adjusted to reflect year-2015 values. The table reports in parentheses the standard errors below the estimates and the p-values below the KS test statistics

Lognormal	$\hat{\mu }$	$\hat{\sigma }$				Log L	BIC	KS test
	2.3548	0.5253				225.3566	459.2666	0.0612
	(0.0619)	(0.0438)						(0.9350)
Gamma	$\hat{k}$	$\hat{\theta }$				Log L	BIC	KS test
	3.3735	3.6486				231.4724	471.4982	0.1033
	(0.5375)	(0.6269)						(0.3993)
Mixture	$\hat{\alpha }_1$	$\hat{\alpha }_2$	$\hat{k}_1$	$\hat{k}_2$	$\hat{\theta }$	Log L	BIC	KS test
	0.9861	0.0139	5	33	2.2840	221.7991	464.9815	0.0700
	(0.2028)	(0.0020)			(0.1561)			(0.8478)

**Fig. 1:**
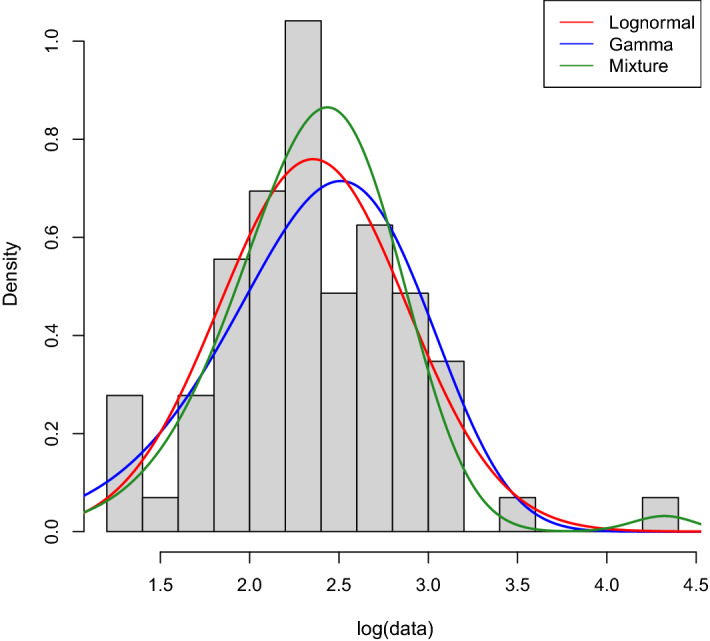
Histogram of the log-transformed data for Sample $A'$ with the addition of the theoretical curves. The losses in Sample $A'$ are adjusted to reflect year-2015 values

The choice of a liability data set that spans a period different from that of the asset log-returns and quite far in time is mainly due to the lack of data sets made available by insurance companies. Our adjustments to the data set *danishuni* assume the independence between liabilities and log-returns. The representativeness of the adjusted data set could be limited since there is no evidence that more recent liability data sets have similar characteristics, but we think that working with simulated losses would be less realistic than using liabilities originated from observed data. The period January 2010 - December 2020, chosen to compute the daily log-returns, has seen a growth of the capital markets if we exclude year 2010 (when the sovereign debt crisis in Europe was still ongoing), Black Monday 2011 (which refers to the date August 8, 2011, when U.S. and global stock markets crashed in the aftermath of the credit rating downgrade of the U.S. sovereign debt by Standard and Poor’s), and year 2020 (when the COVID-19 outbreak started). Referring to this period, portfolios designed to minimize capital requirements could have significant allocations in assets with higher returns. As a consequence, optimal portfolios are generally composed of the index S&P 500 and asset LQD, and only in a few cases they also include the less risky asset SHY.

### Parameter estimation for the liability distributions

**Fig. 2:**
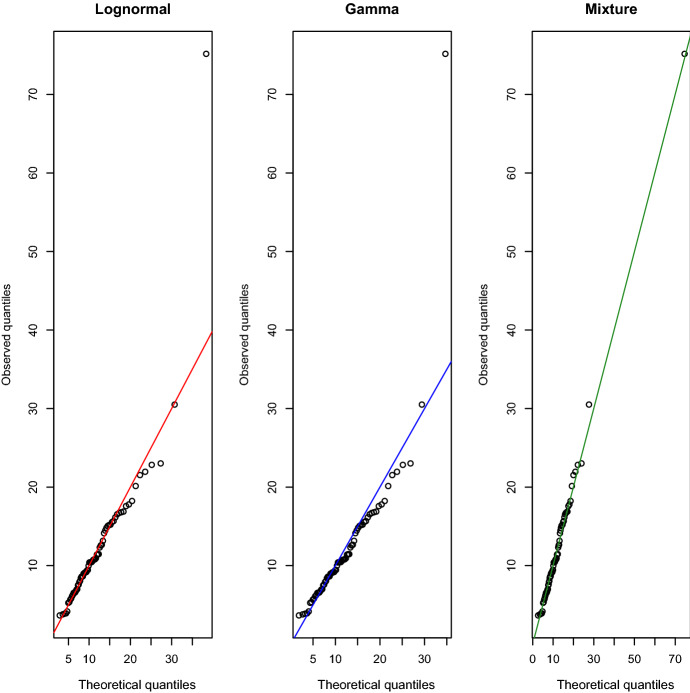
Q-Q plots of the three theoretical distributions for Sample $A'$. The losses in Sample $A'$ are adjusted to reflect year-2015 values

We use the maximum likelihood estimator (MLE) for the three theoretical distributions considered. While applying the MLE to the lognormal and gamma distributions is a standard task, it is less straightforward for a mixture of Erlang distributions with a common scale parameter. To this aim, we use the approach proposed by Lee and Lin (2010), who developed a modified expectation-maximization (EM) algorithm tailored to the class of mixtures of Erlang distributions with a common scale parameter. Their procedure consists of three parts that are the standard EM algorithm, a parameter initialisation using the approximation of Tijms (1994), and an adjustment and diagnosis of parameters. Table 3 reports the estimation results for Sample $A'$ and, in particular, shows, for each distribution, the estimates with the corresponding standard errors in parenthesis, the log-likelihood value at the estimated vector of parameters, the Bayesian information criterion (BIC), and the Kolmogorov-Smirnov (KS) test. Before applying the MLE to Sample $A'$, we use the annual inflation index to have losses reflecting monetary amounts for year 2015. The *p*-values of the computed KS statistics are in parentheses, and we observe that they do not imply the rejection of any of the three distributions. The modified EM algorithm of Lee and Lin (2010) returns a mixture of only two Erlang distributions for Sample $A'$ after adjusting it to have amounts reflecting year-2015 values. The gamma distribution fits the sample worse than the other two distributions since it has the highest KS statistic, largest negative loglikelihood, and largest BIC. For the lognormal distribution and the mixture of two Erlang distributions, we have contrasting results because the lognormal distribution has a slightly smaller KS statistic but a larger negative loglikelihood. However, the lognormal distribution has a lower BIC than the selected mixture of Erlang distributions, due to the fact that the former has only two parameters and the latter has five. To investigate further the goodness-of-fit of the three distributions and examine the differences in fitting the sample, in Fig. 1 we display the histogram of the log-transformed data with the three theoretical curves. We see that the mixture of two Erlang distributions is the only one to admit values as large as the maximum loss in the sample. Indeed, the last rectangle on the right side of the histogram corresponds to the highest loss, and only the green curve, the one corresponding to the mixture of two Erlang distributions, presents density values that take this rectangle into account. The Q-Q plots in Fig. 2 confirm this result since the mixture of two Erlang distributions displays right-tail quantiles close to the empirical quantile corresponding to the highest loss in the sample.

### CVaR constraint optimization

In this section, we solve the optimization Problem (10)–(13) by Algorithm 1. The algorithm has been implemented in the language and environment for statistical computing R, and it uses the function *simplex* of the R package *glpkAPI* of Gelius-Dietrich (2021) to solve the LP problem at each iteration. The confidence level for the CVaR is $\alpha =99\%$, the standard value imposed by the Swiss Solvency Test. In the implementation of Algorithm 1, we set $\lambda =1000$ and $\epsilon =\text {10E-11}$. The solvency horizon is $\tau =21$ since the observed losses are on a monthly basis. Similarly to Asanga et al. (2014), we apply the following strategy when solving Problem (10)–(13):

1. Compute the sample statistics needed for the moment-matching method of Høyland et al. (2003) and estimate the liability parameters for the three theoretical distributions as explained in Sect. 4.2.
2. Apply the expected premium principle to calculate the insurance premium: $p_t=(1+\eta )\,E[Y_{t,t+\tau }]$, where the relative security loading factor $\eta$ equals 0.1.
3. Generate $m=10000$ scenarios ${\textbf {R}}_{t,t+\tau }(j)$, $j=1,\ldots ,m$, for the asset gross returns by using the moment-matching method of Høyland et al. (2003) to simulate asset log-returns with a monthly time scale and taking the exponential of the simulated log-returns.
4. Solve the optimization problem under the three theoretical distributions for the insurer’s liability and find the optimal required capital $c_t^*$ and optimal portfolio allocations $x_{i,t}^*$, $i=1,2,3$.

#### Efficient frontier analysis

We build efficient frontiers by applying the above strategy for different lower levels, $\gamma$, of the expected ROC. To obtain the solution corresponding to the minimum value of $\gamma$, we solve Problem (10)–(13) without the portfolio performance constraint. The sample statistics used to generate asset log-returns with the moment-matching method of Høyland et al. (2003) are displayed in Table 4, and they are computed by applying formulae (26)–(30) to daily log-returns in Sample *A* with $\tau =21$. The liability parameters are estimated by using Sample $A'$ and by adjusting the losses in order to reflect year-2015 values. These estimates are reported in Table 3.

**Table 4:** Sample statistics computed with formulae (26)–(30) using daily log-returns in Sample *A* and $\tau =21$

Asset	Mean	S.D.	Skewness	Kurtosis
S&P 500	0.00821	0.04600	–0.09531	3.20161
SHY	0.00068	0.00245	0.02194	3.12684
LQD	0.00446	0.01622	–0.11963	3.10271
	Correlation	SHY	LQD	
	S&P 500	–0.30348	–0.10616	
	LQD	0.54418		

**Fig. 3:**
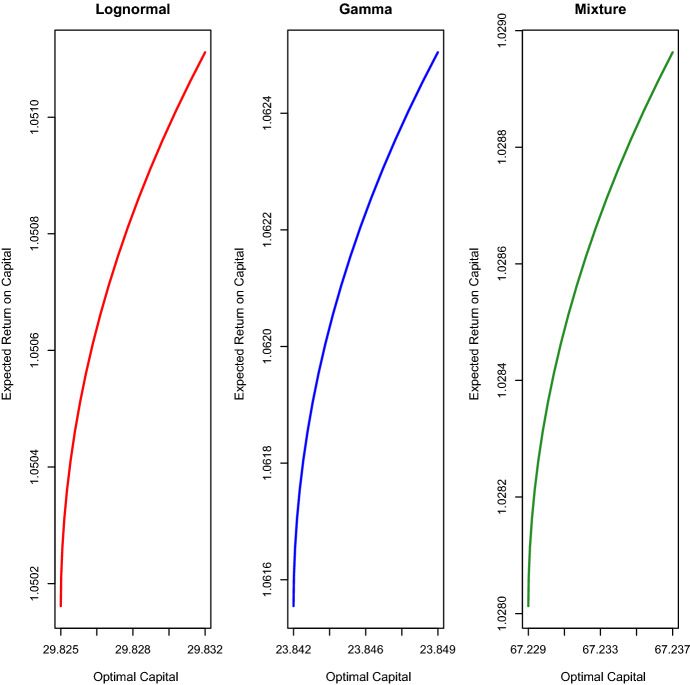
Efficient frontiers built under the three theoretical distributions for Sample $A'$. The losses in Sample $A'$ are adjusted to reflect year-2015 values

**Fig. 4:**
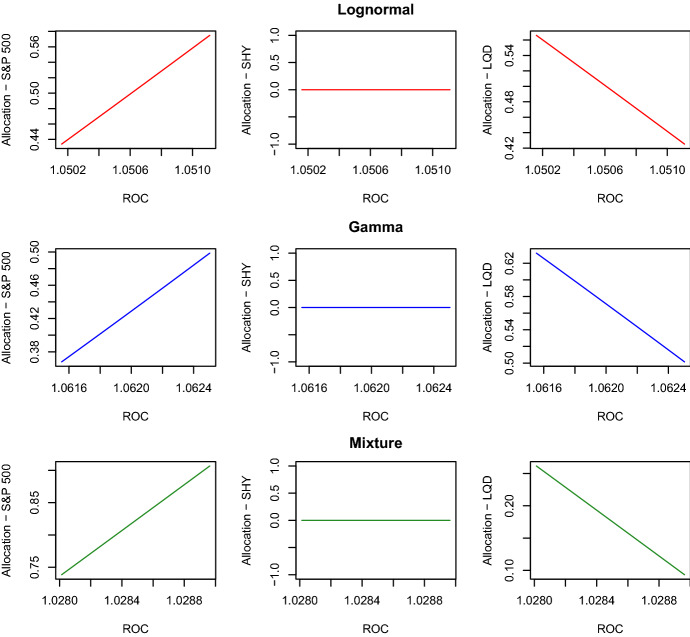
Optimal portfolio allocations under the three theoretical distributions for Sample $A'$. The losses in Sample $A'$ are adjusted to reflect year-2015 values

We analyse the behaviour of the optimal capital required $c_t^*$ and optimal portfolio allocations $x_{i,t}^*$, $i=1,2,3$, under the three distributions. In Fig. 3, we plot the three efficient frontiers and observe that the optimal required capital $c_t^*$ differs among the three distributions. In particular, the mixture of two Erlang distributions implies levels of $c_t^*$ much higher than those of the other two distributions. This result is reasonable because, contrary to the other two distributions, the mixture of two Erlang distributions captures better the losses close to the maximum value present in the sample. The liability distribution also has an impact on the expected ROC. We see that, under the mixture of two Erlang distributions, the expected ROC values are smaller than those obtained under the other two distributions. To complete this efficient frontier analysis, we plot in Fig. 4 the portfolio allocations for the three liability distributions. We see that all the three distributions do not provide investment in the asset SHY but invest the available capital in the other two assets. Specifically, we notice that, as the expected ROC level increases, the S&P 500 allocations increase, and the LQD allocations decrease.

**Fig. 5:**
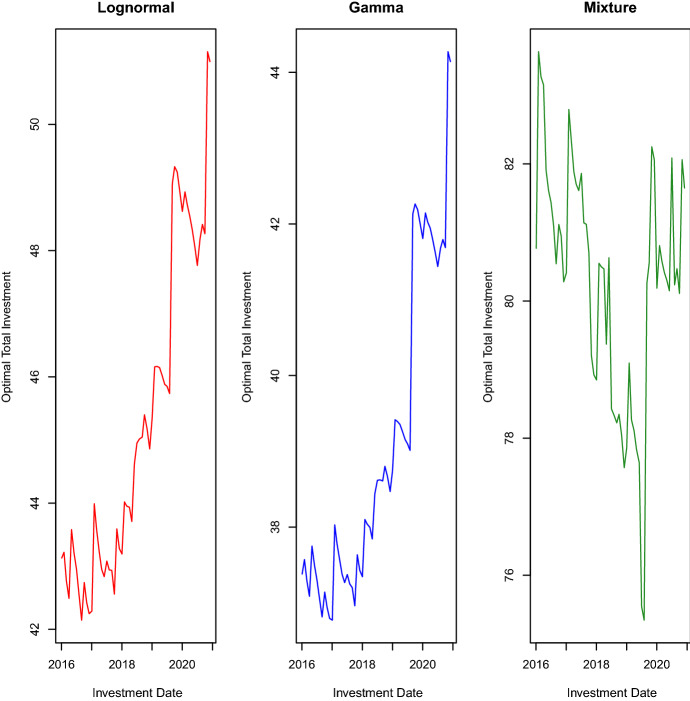
Optimal total investments $p_{t+(\psi -1)\tau }+c_{t+(\psi -1)\tau }^*$, $\psi =1,\ldots ,\Psi$

**Fig. 6:**
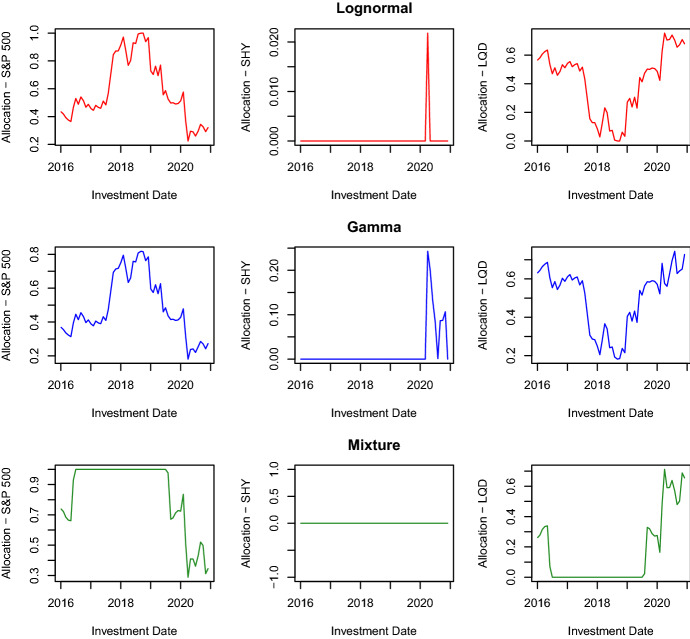
Optimal asset allocations

#### Out-of-sample analysis

We perform an out-of-sample analysis that relies on a rolling-window approach for the asset log-returns and an expanding-window approach for the insurer’s losses when accomplishing a new optimization. In this way, we execute an out-of-sample analysis that differs from Asanga et al. (2014), who apply a rolling-window approach even for the insurer’s liabilities. We do not exclude any observed liability when estimating loss-distribution parameters because we think that large losses are rare and a rolling-window approach could underestimate their reappearance. In Appendix C, we perform an out-of-sample analysis that uses a rolling-window approach for the insurer’s liabilities. This further analysis highlights how the large-loss underestimation is concrete when applying a rolling-window approach.

The rolling-window length corresponds to six years of daily observations. Using Samples *A* and $A'$, we first compute the optimal solutions $(c_t^*,{\textbf {x}}_t^*)$ for the period $[t,t+\tau ]$ by applying Steps 1-4 detailed above. Then, we build a new sample for asset log-returns by eliminating the observations of the first month in Sample *A* and including those of the first month in Sample *B*. In this way, we carry out a monthly portfolio rebalancing. Regarding the insurer’s liabilities, we build the new sample by not excluding any observation from Sample $A'$ and including the first observation from Sample $B'$. Furthermore, we adjust the losses of this new sample to reflect values of the year of the last loss, which is 2016 in this case. Given the new samples for asset log-returns and losses, we recompute the new optimal solutions $(c_{t+\tau }^*,{\textbf {x}}_{t+\tau }^*)$ for the next period by applying Steps 1-4. Repeating the sample and optimization procedures up to the end of Samples *B* and $B'$, we end up with the collection of optimal solutions $(c_{t+(\psi -1)\tau }^*,{\textbf {x}}_{t+(\psi -1)\tau }^*)$, $\psi =1,\ldots ,\Psi$, where $\Psi$ is the length of Sample $B'$. To avoid optimization problems with no feasible solution, we do not set a lower bound for the expected ROC. Figures 5 and 6 and Table 5 display the results.

Figure 5 shows how the optimal total investment $p_t+c_t^*$ evolves over time. The lognormal and gamma distributions have a very similar evolution even though the lognormal distribution usually requires, on any investment date, an optimal investment that is larger than that of the gamma distribution. The mixture of Erlang distributions displays an evolution that differs from those of the other two distributions. In detail, the optimal total investment is much higher on each investment date, and its variation between two consecutive months has a more pronounced intensity even though it is in the same direction showed by the graphs of the other two distributions. Figure 6 shows the evolutions of the optimal allocations $x_{i,t}^*$, $i=1,2,3$. For the lognormal and gamma distributions, we observe that the optimal portfolios invest only in the two assets S&P 500 and LQD. To be precise, there are a few exceptions in 2020. Indeed, there is one date where the lognormal distribution invests a small percentage of the optimal total investment in the asset SHY and some dates where it is the gamma distribution to put money into the asset SHY. The mixture distribution never invests in the asset SHY, and there is a long period of time, from July 2016 to July 2019, with investments only in the asset S&P 500. These results collide with those of Asanga et al. (2014) that instead found, in their out-of-sample analysis, significant allocations in the asset SHY. To justify this difference in the results, we observe that we work with prices on a different period, use a different loss data set, and generate asset log-returns with a different model. We think that what makes the real difference in the two experiments is the way of generating asset log-returns. Asanga et al. (2014) chose to model asset log-returns with three MV-GARCH models by ignoring the mean effect present in the models. On the contrary, we use a moment-matching method that does not ignore the asset means. Likely, this dissimilarity in modelling asset means could be the reason for such different results. To confirm this explanation of the difference in the two analyses, we performed some experiments (results are available on request) where we generated asset log-returns with a DCC-GARCH model under the assumption of zero mean for each asset, and we obtained that most of the optimal capital was allocated in the asset SHY.

Table 5 reports some statistics about the realized insurer’s wealth at the end of each month from January 2016 to December 2020 under the three liability distributions. That is, the table gives summary statistics for the differences

$$\begin{aligned} {\textbf {r}}'_\psi {\textbf {z}}_{\psi -1}^*-y_\psi ,\quad \psi =1,\ldots ,\Psi , \end{aligned}$$

where ${\textbf {r}}_\psi$ is the vector of the realized asset gross returns in the $\psi$th month, $y_{\psi }$ is the realized insurer’s liability in the $\psi$th month, and ${\textbf {z}}_{\psi -1}^*$ is the vector containing the optimal amounts invested in each asset at the beginning of the $\psi$th month. It is worth highlighting that only the mixture distribution ensures a positive insurer’s wealth in each month. Indeed, for the other two distributions, there are two dates where the insurer’s wealth is negative and the insurer faces bankruptcy.

**Table 5:** Statistics about the realized insurer’s wealth at the end of each month from January 2016 to December 2020

Distribution	Min.	Max.	Mean	S.D.	Skewness	Kurtosis
Lognormal	–17.78315	43.62607	29.32046	10.36708	–2.74788	12.96112
Gamma	–24.52729	36.87083	23.05091	10.28401	–2.88796	13.69663
Mixture	11.95836	77.69185	64.75780	11.30090	–2.96548	13.97982

## Conclusions

In this article, we extend the optimization problem with CVaR constraint of Asanga et al. (2014) to any integrable liability distribution. To solve the problem, we propose applying the Kelley-Cheney-Goldstein algorithm, and we give proof of the convergence of the algorithm. We adopt three distributions for modelling the insurer’s liability: the lognormal distribution, the gamma distribution, and a mixture of Erlang distribution with a common scale parameter. The results show that the choice of the liability distribution has a strong impact on the optimal capital and asset allocations. Specifically, the mixture distribution offers a higher protection for what concerns our data set of losses since, contrary to the other two distributions, in the out-of-sample analysis the insurer’s wealth never becomes negative at the end of a month. Clearly, this higher protection is at the cost of a greater optimal capital. The optimal allocations of our experiments usually do not include the asset SHY, which is the least risky because it is a fund investing in Treasury Bonds with a maturity from 1 to 3 years. When working with the mixture distribution, we often obtain that the optimal investment should be just in the asset S&P 500. Our results differ from those of Asanga et al. (2014), who found significant allocations in the asset SHY. This difference in the results is probably due to the mean effect in modelling asset log-returns because the analysis in Asanga et al. (2014) ignores it, whereas our empirical application does not. As discussed in Sect. 4.1, the almost total absence of the asset SHY in the optimal portfolios is mainly due to the chosen period from January 2010 to December 2020, which was a period of growth for capital markets. Hence, optimal portfolios minimizing the capital requirement tend to contain significant allocations in the assets with higher returns. Different periods characterized by little growth and turbulence in the markets, like the one used by Asanga et al. (2014), should return optimal portfolios with allocations in the less risky assets.

Future developments of the optimization problem proposed in this article could regard its application with other liability distributions and asset log-return models. For instance, it may be applied in the case of huge losses when the insurer’s liability is modelled with a generalized Pareto distribution, which is a distribution that finds justification in the Extreme Value Theory (EVT) (see, e.g., Embrechts et al. 2013). Regarding the asset log-return models, it would be interesting to consider as future research alternative models like those proposed by Mudry and Paraschiv (2016) and Koliai (2016) that use the EVT to model the marginal distributions of the returns and copula functions to capture the dependence structures. Finally, other developments could go towards the definition of multistage models that give the possibility of changing the asset allocations at intermediate dates.

## Figures and Tables

**Fig. 7:**
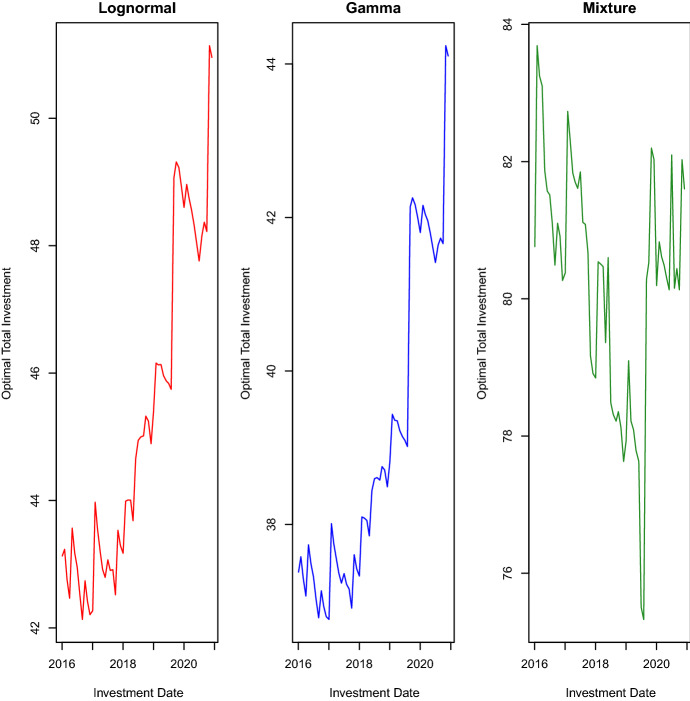
Optimal total investments $p_{t+(\psi -1)\tau }+c_{t+(\psi -1)\tau }^*$, $\psi =1,\ldots ,\Psi$

**Fig. 8:**
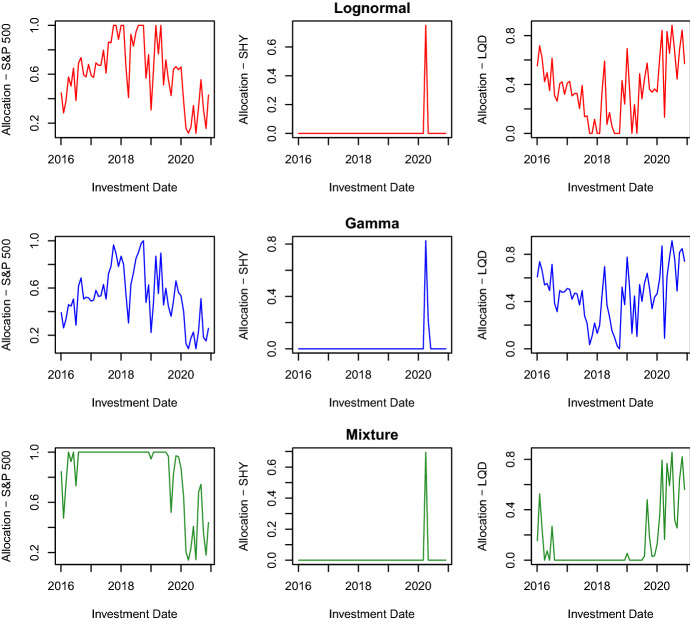
Optimal asset allocations

**Fig. 9:**
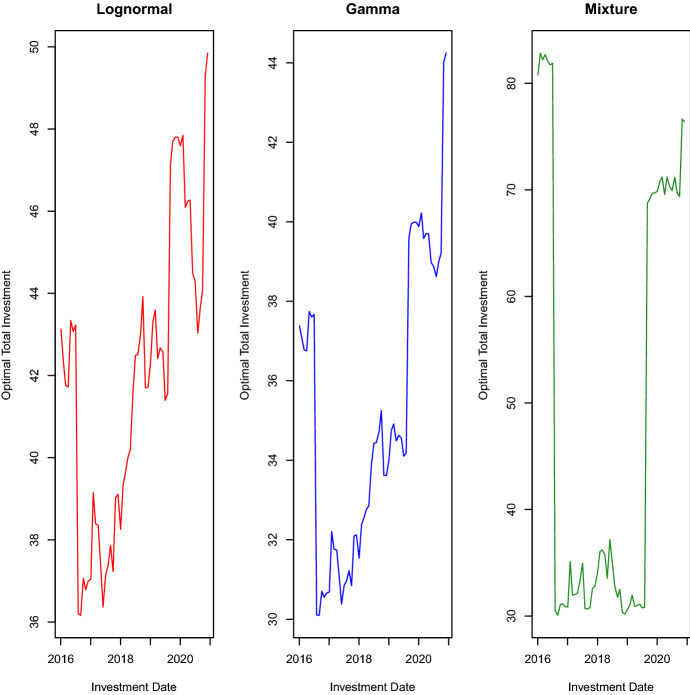
Optimal total investments $p_{t+(\psi -1)\tau }+c_{t+(\psi -1)\tau }^*$, $\psi =1,\ldots ,\Psi$

**Fig. 10:**
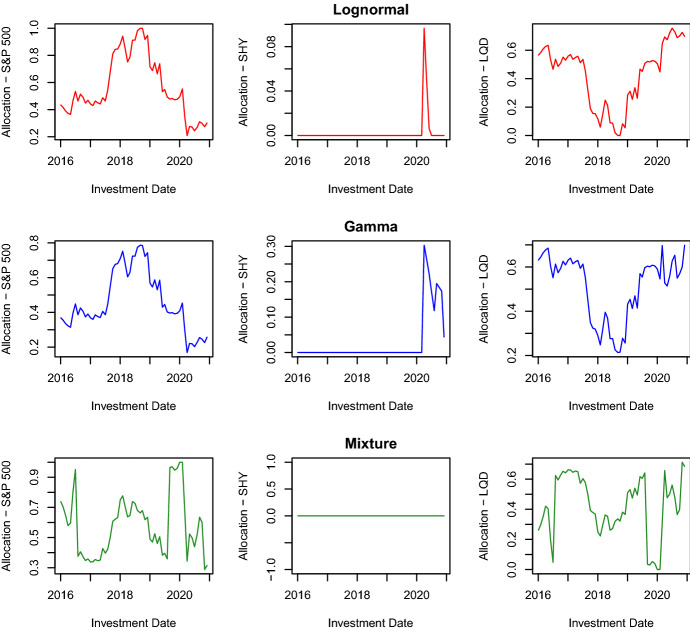
Optimal asset allocations

## Appendix A

In this Appendix, we report the definitions of the three probability distributions used to describe the insurer’s liability.

Lognormal distribution.: The r.v. *Y* is lognormal distributed with parameters $\mu \in \mathbb {R}$ and $\sigma >0$ if the transformation $X=\ln {Y}$ is normally distributed with mean $\mu$ and standard deviation $\sigma$. Then, the density function of *Y* is given by $$\begin{aligned} f_Y(y)=\frac{1}{y\sigma \sqrt{2\pi }}\exp \left( -\frac{(\ln (y)-\mu )^2}{2\sigma ^2}\right) ,\quad y>0. \end{aligned}$$
Gamma distribution.: The r.v. *Y* is gamma distributed with shape parameter $\beta >0$ and scale parameter $\theta >0$ if it has the density function $$\begin{aligned} f_Y(y)=\frac{y^{\beta -1}e^{-y/\theta }}{\theta ^\beta \Gamma (\beta )},\quad y>0, \end{aligned}$$ where $\Gamma (\beta )$ denotes the gamma function evaluated at $\beta$: $$\begin{aligned} \Gamma (\beta )=\int _0^{+\infty }t^{\beta -1}e^{-t}dt. \end{aligned}$$
Mixture of Erlang distributions with a common scale parameter.: We recall that an Erlang distribution is a gamma distribution whose shape parameter $\beta$ is a positive integer. The r.v. *Y* is a mixture of $\varpi$ Erlang distributions with a common scale parameter $\theta >0$ if it has the density function $$\begin{aligned} f_Y(y)=\sum _{i=1}^\varpi \alpha _i\frac{y^{\beta _i-1}e^{-y/\theta }}{\theta ^{\beta _i}(\beta _i-1)!},\quad y>0, \end{aligned}$$ where $\alpha _1,\ldots ,\alpha _\varpi$ are positive real numbers that sum up to 1, and $\beta _1,\ldots ,\beta _\varpi$ are positive integers.

## Appendix B

In this appendix, we model daily log-returns as in Asanga et al. (2014), who used the class of DCC-GARCH processes introduced by Engle (2002). Asanga et al. (2014) evaluated the performance of the CVaR optimization problem under three multivariate GARCH models, i.e., the DCC model of Engle (2002), the conditional constant correlation model of Bollerslev (1990), and a model with uncorrelated assets. Their results confirm the superiority of the DCC model of Engle (2002).

**Table 6:** Parameters estimates with standard errors in parentheses for the DCC-GARCH model when using daily log-returns of the assets S&P 500, SHY, and LQD observed from January 2010 to December 2015 (Sample *A*)

Estimation stage	Model parameters			
Univariate Garch	Asset	$\omega$	$\alpha$	$\beta$
	S&P 500	3.95E-06	0.1385	0.8214
		(7.84E-06)	(0.0241)	(0.0645)
	SHY	4.66E-09	0.0641	0.9038
		(1.92E-07)	(0.0137)	(0.0205)
	LQD	2.74E-07	0.0449	0.9321
		(5.71E-07)	(0.0201)	(0.0216)
DCC		$\theta _1$	$\theta _2$	
		0.0378	0.8898	
		(0.0131)	(0.0374)	

According to the DCC model, asset daily log-returns follow the process

B1 $$\begin{aligned} \log {{\textbf {R}}_{t+1}}={\textbf {m}}_{t+1}+\varvec{\varepsilon }_{t+1}, \end{aligned}$$

where ${\textbf {m}}_{t+1}$ is the *n*-dimensional $\mathcal {F}_t$-measurable conditional mean log-return vector, and $\varvec{\varepsilon }_{t+1}=(\varepsilon _{1,t+1}\ldots ,\varepsilon _{n,t+1})^T$ has a conditionally multivariate normal distribution with mean ${\textbf {0}}$ and covariance matrix $H_{t+1}$, that is, $\varvec{\varepsilon }_{t+1}\vert \mathcal {F}_t\sim N({\textbf {0}},H_{t+1})$. The DCC structure allows us to separate the dynamics for the time-varying conditional variances of each asset from the dynamics for the time-varying conditional correlation matrix. Specifically, we have

B2 $$\begin{aligned} H_{t+1}=&\,D_{t+1}^{1/2}\Sigma _{t+1}D_{t+1}^{1/2},\nonumber \\ D_{t+1}=&\,\text {diag}(h_{1,t+1},\ldots ,h_{n,t+1}),\nonumber \\ \Sigma _{t+1}=&\,\text {diag}(q_{11,t+1}^{-1/2},\ldots ,q_{nn,t+1}^{-1/2})\,Q_{t+1}\,\text {diag}(q_{11,t+1}^{-1/2},\ldots ,q_{nn,t+1}^{-1/2}),\nonumber \\ Q_{t+1}=&\,(1-\theta _1-\theta _2)\overline{Q}+\theta _1{\textbf {u}}_t{\textbf {u}}_t^T+\theta _2Q_t, \end{aligned}$$

where the elements of the $n\times n$ diagonal matrix $D_{t+1}$ follow the standard GARCH(1,1) processes

B3 $$\begin{aligned} h_{i,t}=\omega _i+\alpha _i\varepsilon _{t-1}^2+\beta _ih_{i,t-1},\quad i=1,\ldots ,n. \end{aligned}$$

The matrix $\Sigma _{t+1}$, whose element in the *i*th row and *j*th column is $\rho _{ij,t+1}=q_{ij,t+1}q_{ii,t+1}^{-1/2}q_{jj,t+1}^{-1/2}$, represents the time-varying conditional correlation matrix of the log-return vector ${\textbf {R}}_{t+1}$. The matrix $Q_{t+1}$, formed by elements $q_{ij,t+1}$, for $i,j=1,\ldots ,n$, follows the GARCH(1,1) process specified in (B2), where the process ${\textbf {u}}_t$ is the $n\times 1$ vector of devolatilized but correlated innovations (i.e., $u_{i,t}=h_{i,t}^{-1/2}\varepsilon _{i,t}$), and $\overline{Q}$ is the unconditional covariance matrix of ${\textbf {u}}_t$. We assume that the conditions required for covariance stationarity and positive definiteness of $H_{t+1}$, for any *t*, are satisfied by the univariate GARCH parameters in (B3), $\omega _i$, $\alpha _i$, and $\beta _i$, and by the DCC parameters in (B2), $\theta _1$ and $\theta _2$.

To estimate the parameters of a DCC model, first we need to consider the specification of the conditional mean vector ${\textbf {m}}_{t+1}$ in Eq. (B1). Even though there are many ways to accomplish this task, we set ${\textbf {m}}_{t+1}$ simply equal to the vector of sample means to see how time-varying conditional variances and correlations affect capital requirements and portfolio allocations in comparison with the moment-matching method of Høyland et al. (2003). For the other model parameters, we follow the two-stage maximum likelihood estimator (MLE) algorithm proposed by Engle and Sheppard (2001). Then, in the first stage, the univariate GARCH parameters are estimated by maximizing the log-likelihood function

B4 $$\begin{aligned} \log {L}=-\frac{1}{2}\sum _{k=1}^K\left( \log {\vert H_k\vert }+\varvec{\varepsilon }_k^TH_k^{-1}\varvec{\varepsilon }_k\right) , \end{aligned}$$

where *K* is the sample size and the conditional correlation matrix of ${\textbf {R}}_k$, $\Sigma _k$, is assumed to be the identity matrix. In the second stage, the DCC parameters are estimated by maximizing the log-likelihood function (B4) with the correct specification of $\Sigma _k$. It means that, in the second stage, only the parameters $\theta _1$ and $\theta _2$ are estimated. To perform this two-stage estimation procedure, we use the function *dccfit* of the R package *rmgarch* of Ghalanos (2019). Table 6 reports the estimates for Sample *A*. We remark that all the three univariate series display a high degree of persistence, being $\alpha +\beta =0.9599$ for S&P 500, $\alpha +\beta =0.9679$ for SHY, and $\alpha +\beta =0.9770$ for LQD. A similar persistence level holds for the DCC parameters since $\theta _1+\theta _2=0.9276$.

In this appendix, we display only the results of the out-of-sample analysis detailed in Sect. 4.3.2. To solve Problem (10)–(13), we apply the strategy laid out in Sect. 4.3 by generating monthly log-return scenarios with a DCC model. Figure 7 displays the evolutions of the optimal total investments for the three liability distributions from January 2016 to December 2020. We observe that they are very similar to those in Fig. 5, where we use the moment-matching method of Høyland et al. (2003). Figure 8 shows how the portfolio allocations evolve for the three liability distributions. As in Fig. 6, we observe that the investment in the asset SHY is almost always zero and the mixture of Erlang distributions puts all the total investment into the index S&P 500 in many months.

## Appendix C

In this appendix, we show an out-of-sample analysis where, as in Asanga et al. (2014), we apply a rolling-window approach to both the asset log-returns and the insurer’s losses. Hence, this analysis is different from the out-of-sample analysis in Sect. 4.3.2, where we apply an expanding-window approach to the insurer’s losses.

The rolling-window length is six years. We use Samples *A* and $A'$ to compute the optimal solutions $(c^*_t,{\textbf {x}}^*_t)$ for the period $[t,t+\tau ]$ by applying Steps 1-4 detailed in Sect. 4.3. Then, to build the new sample of asset log-returns, we eliminate the observations in the first month of Sample *A* and include those in the first month of Sample *B*, while, to build the new sample of losses, we eliminate the first observation in Sample $A'$ and include the first one in Sample $B'$. The new sample of losses is adjusted to reflect values of the year of the last loss, which is 2016 in this case. Observe that we are carrying out a monthly portfolio rebalancing. Given the new samples for asset log-returns and losses, we recompute the new optimal solutions $(c^*_{t+\tau },{\textbf {x}}^*_{t+\tau })$ for the next period by applying Steps 1-4 of Sect. 4.3. Repeating the sample and optimization procedures up to the end of Samples *B* and $B'$, we end up with the collection of optimal solutions $(c_{t+(\psi -1)\tau }^*,{\textbf {x}}_{t+(\psi -1)\tau }^*)$, $\psi =1,\ldots ,\Psi$, where $\Psi$ is the length of Sample $B'$. As in Sect. 4.3.2, to avoid optimization problems with no feasible solution, we do not set a lower bound for the expected ROC. Figure 9 displays the evolution of the optimal total investment $p_t+c^*_t$, Fig. 10 the evolutions of the optimal total allocations $x^*_{i,t}$, $i=1,2,3$, and Table 7 the statistics about the realized insurer’s wealth at the end of each month.

Looking at Fig. 9, we observe that, for all the three distributions, there is a drop in optimal total investment around mid-2016. Indeed, a significant loss is no longer in the sample, and the optimization problems return optimal capital requirements smaller than the expanding-window approach. This evidence does not characterize Fig. 5, where the expanding-window approach holds this significant loss, and the optimization problems provide optimal capital requirements not too different from the previous ones. An upward jump occurs in year 2019. Indeed, a significant loss has entered the sample so that the optimization problems return higher capital requirements. We can also observe the entry of this large loss into the sample in Fig. 5, which entails a significant jump for all the three distributions.

**Table 7:** Statistics about the realized insurer’s wealth at the end of each month from January 2016 to December 2020

Distribution	Min.	Max.	Mean	S.D.	Skewness	Kurtosis
Lognormal	–21.96963	41.81310	25.94025	10.86834	–2.47579	11.58756
Gamma	–29.37180	36.64244	19.09946	10.84130	–2.35865	11.20332
Mixture	–32.74864	72.90299	32.45823	24.35307	0.22866	2.22001

Turning our attention to Fig. 10 and looking at the panel of the mixture of Erlang distributions, we can observe how the rolling-window approach substantially modifies the optimal portfolio allocations with respect to the expanding-window approach. Indeed, removing a significant loss from the samples determines optimal portfolios that do not invest, from mid-2016 to the end of 2019, all the capital in the index S&P 500 as, on the contrary, displayed in Fig. 6. The rolling-window approach applied to the adjusted data set considerably modifies the realized insurer’s wealth obtained with the mixture of Erlang distributions as evidenced by the statistics displayed in Table 7. Indeed, compared with the statistics reported in Table 5, the minimum wealth realized with the mixture of Erlang distributions is now negative like the other two distributions, and it is actually the smallest one. Moreover, the standard deviation is higher than the one reported in Table 5 implying that, in this case, the realized insurer’s wealth is more dispersed.

The differences between the two out-of-sample analyses show how the mixture of Erlang distributions is more effective when fitting data sets including large losses and, in this case, it permits to compute accurate capital requirements. Otherwise, in the absence of large losses in the data sets, such a mixture could underestimate the reappearance of significant losses.

## Footnotes

1 Even though the following result holds under more general dependence structures, we assume that the r.v. $Y_{t,t+\tau }$ is independent of the $\sigma$-algebra $\mathcal {F}_t\bigvee \sigma \left( {\textbf {R}}_{t,t+\tau }\right)$.

## References

[CR1] Acerbi C, Tasche D (2002) On the coherence of expected shortfall. J Bank Financ 26(7):1487–1503

[CR2] Artzner P (1999) Application of coherent risk measures to capital requirements in insurance. North Am Actuar J 3(2):11–25

[CR3] Artzner P, Delbaen F, Eber J-M, Heath D (1999) Coherent measures of risk. Math Financ 9(3):203–228

[CR4] Asanga S, Asimit A, Badescu A, Haberman S (2014) Portfolio optimization under solvency constraints: a dynamical approach. North Am Actuar J 18(3):394–416

[CR5] Asimit AV, Badescu AM, Siu TK, Zinchenko Y (2015) Capital requirements and optimal investment with solvency probability constraints. IMA J Manag Math 26(4):345–375

[CR6] Balbás A (2008) Capital requirements: are they the best solution? Tech. rept. Universidad Carlos III de Madrid, Departamento de Economía de la Empresa

[CR7] Bollerslev T (1990) Modelling the coherence in short-run nominal exchange rates: a multivariate generalized ARCH model. Rev Econom Stat 72(3):498–505

[CR8] Dhaene J, Vanduffel S, Goovaerts MJ, Kaas R, Tang Q, Vyncke D (2006) Risk measures and comonotonicity: a review. Stoch Model 22(4):573–606

[CR9] Directive 2009/138/EC of the European Parliament and of the Council of 25 November 2009 on the taking-up and pursuit of the business of Insurance and Reinsurance (Solvency II), Official Journal of the European Union, L 335/1. 17.12.2009

[CR10] Dutang C, & Charpentier A (2020) CASdatasets: insurance datasets. R package version 1.0-11 (11-12-2020) available at http://cas.uqam.ca

[CR11] Embrechts P, Klüppelberg C, Mikosch T (2013) Modelling extremal events: for insurance and finance, vol 33. Springer Science & Business Media, USA

[CR12] Engle R (2002) Dynamic conditional correlation: A simple class of multivariate generalized autoregressive conditional heteroskedasticity models. J Bus Econom Stat 20(3):339–350

[CR13] Engle R, Sheppard K (2001) Theoretical and empirical properties of dynamic conditional correlation multivariate GARCH. National Bureau of Economic Research, Cambridge, MA

[CR14] Farkas W, Koch-Medina P, Munari C (2015) Measuring risk with multiple eligible assets. Math Financ Econ 9(1):3–27

[CR15] Federal Office of Private Insurance (FOPI) (2004) White paper of the Swiss Solvency Test. Bern, Switzerland

[CR16] Gelius-Dietrich G (2021) glpkAPI: R Interface to C API of GLPK. R package version 1(3):3

[CR17] Ghalanos A (2019) rmgarch: Multivariate GARCH models. R package version 1.3-7

[CR18] Høyland K, Kaut M, Wallace SW (2003) A heuristic for moment-matching scenario generation. Comput Optim Appl 24(2):169–185

[CR19] Kaas R, Goovaerts M, Dhaene J, Denuit M (2008) Modern actuarial risk theory: using R, vol 128. Springer Science & Business Media, UK

[CR20] Kaucic M, Daris R (2015) Multi-Objective stochastic optimization programs for a non-life insurance company under solvency constraints. Risks 3(3):390–419

[CR21] Kelley JE (1960) The cutting-plane method for solving convex programs. J Soc Ind Appl Math 8(4):703–712

[CR22] Koliai L (2016) Extreme risk modeling: An EVT-pair-copulas approach for financial stress tests. J Bank Financ 70:1–22

[CR23] Krokhmal P, Palmquist J, Uryasev S (2002) Portfolio optimization with conditional value-at-risk objective and constraints. J Risk 4:43–68

[CR24] Lee SCK, Lin XS (2010) Modeling and evaluating insurance losses via mixtures of Erlang distributions. North Am Actuar J 14(1):107–130

[CR25] Lee SCK, Lin XS (2012) Modeling dependent risks with multivariate Erlang mixtures. ASTIN Bull J IAA 42(1):153–180

[CR26] Mankai S, Bruneau C (2012) Optimal economic capital and investment: decisions for a non-life insurance company. Bankers Markets & Investors: an Acad Profess Rev 119:19–30

[CR27] Mudry PA, Paraschiv F (2016) Stress-Testing for Portfolios of Commodity Futures with Extreme Value Theory and Copula Functions. Computational Management Science, Springer, UK

[CR28] Rockafellar RT, (1970) Convex analysis, vol 18. Princeton University Press, UK

[CR29] Rockafellar RT, Uryasev S (2000) Optimization of conditional value-at-risk. J Risk 2:21–41

[CR30] Rockafellar RT, Uryasev S (2002) Conditional value-at-risk for general loss distributions. J Bank Financ 26(7):1443–1471

[CR31] Ryan JA, & Ulrich, JM 2022. quantmod: Quantitative Financial Modelling Framework. R package version 0.4.20

[CR32] Tijms HC (1994) Stochastic models: an algorithmic approach. John Wiley & Sons, USA

[CR33] Verbelen R, Gong L, Antonio K, Badescu A, Lin S (2015) Fitting mixtures of Erlangs to censored and truncated data using the EM algorithm. ASTIN Bull J IAA 45(3):729–758

[CR34] Willmot GE, Lin XS (2011) Risk modelling with the mixed Erlang distribution. Appl Stoch Model Bus Ind 27(1):2–16

